# Using Physical Activity to Enhance Health Outcomes Across the Life Span

**DOI:** 10.3390/jfmk5010002

**Published:** 2020-01-04

**Authors:** Dev Roychowdhury

**Affiliations:** DR ACADEMY, Melbourne, VIC 3000, Australia; info@drdevroy.com

**Keywords:** physical activity, health, life span, psychology, physiology, kinesiology

## Abstract

Physical activity has been widely recognized as one of the primary determinants that proliferates positive psychophysiological health in individuals. Despite the numerous benefits of engaging in physical activity, a majority of the global population continues to be physically inactive or sedentary. The aim of this brief commentary is to capture the benefits of engaging in regular physical activity across the life span. In particular, this paper will highlight the benefits of engaging in regular physical activity with respect to age, gender, atypical populations, and lifestyle. Future research and recommendations have also been addressed.

## 1. Introduction

Despite the well-established benefits of engaging in regular physical activity [1,2], recent trends have revealed that a large proportion of the global population is increasingly becoming sedentary [3,4], which has been associated with increased risks for several chronic conditions and mortality [5,6,7]. The global economic cost of physical inactivity has been conservatively estimated at $67.5 billion [8,9] with no improvements in the levels of physical inactivity recorded in the past decade [10]. Understandably, the emotional and psychological burden are likely to be far reaching and horrid. 

Physical activity has been defined as “any bodily movement produced by skeletal muscles that requires energy expenditure” [11]. The World Health Organization (WHO) has developed global recommendations on physical activity for health with the primary aim of guiding policy makers on the requisite amount of physical activity with respect to its frequency, duration, intensity, type, and total amount [12]. The recommendations that are set out address three age groups: 5–17 years old; 18–64 years old; and 65 years old and above [12]. It is primarily recommended that children and youth aged 5–17 years ought to engage in at least 60 min of moderate to vigorous intensity physical activity per day; adults aged 18–64 years ought to do at least 150 min of moderate intensity aerobic physical activity on a weekly basis or do at least 75 min of vigorous intensity aerobic physical activity in a week or an equivalent combination of moderate and vigorous intensity activity; and adults aged 65 years and above ought to do at least 150 min of moderate intensity aerobic physical activity in a week or do at least 75 min of vigorous intensity aerobic physical activity on a weekly basis or an equivalent combination of moderate and vigorous intensity activity [13]. 

Despite the clear practical recommendations that exist and explosion of research reporting on the benefits of engaging in regular physical activity, recent research now suggests that it is no longer sufficient to simply meet these recommendations to address health needs and lower health risks [14]. Furthermore, the conceptualization of the terms ‘physical inactivity’ and ‘sedentary behaviour’ has also been controversial [15,16], which has implications for the way research protocols are designed and policy guidelines are formulated. Future research should, therefore, attempt to focus on arriving at a clear understanding of and consensus on what these terms constitute along with their theoretical, observational, and practical distinctions. For the purpose of this paper, the terms physical inactivity and sedentary behaviours are used interchangeably. 

It is widely accepted that a range of factors affect individuals’ involvement in physical activity, including psychological, social, environmental, and administrative factors [17]. Given the significance of different factors and the role they play in individuals’ final decision to undertake and maintain regular physical activity [18], it is imperative to understand how motivational factors aid engagement in physical activity within such complex and multi-dimensional contexts. Research on participation motivation has clearly indicated that individuals’ involvement in specific types of physical activity may be functionally characterized by the primary participation motives they have for engaging in those activities [19,20,21]. Although motivation research pertaining to physical activity has received substantial consideration, other determinants have received scant attention. The purpose of this brief commentary, therefore, is to encapsulate the benefits of engaging in regular physical activity across the life span. More specifically, this paper will highlight the benefits of engaging in regular physical activity with respect to age, gender, atypical populations, and lifestyle.

## 2. Physical Activity across Age Groups 

Movement-based activity has been found to positively affect individuals’ psychophysiology across different life stages. Physical activity has been linked to a number of adaptive psychological, physical, and social outcomes, especially in early childhood (i.e., birth to five years), including lower cardiovascular risk factors [22,23], improved bone development [24,25], decreased fat and body mass indices [26,27,28,29], improved motor development [30], and improved emotional, cognitive, and social development [31,32]. 

Research with school-aged children have also reported that physical activity may improve cognitive functioning and academic performance at school [33,34,35,36,37,38,39]. Physical activity has also been found to improve psychological health and prevent mental health conditions in young people [40,41]. Furthermore, recent neuroimaging research involving children has found that those who participated in a nine-month physical activity program showed reduction in fMRI brain activation in the right anterior prefrontal cortex and improvements in performance on a task of attentional and interference control when compared to the wait-list control group [42]. Similarly, movement-based learning environments have shown to improve children’s executive functioning [43,44,45] and academic performance [46,47,48,49,50,51,52,53]. Studies have further reported that certain core executive functions, that appear to be activated through physical activity, are fundamental for children’s psychophysiological and social development [54] and may play a vital role in their overall academic success [55,56,57]. 

Research on physical activity with adults has revealed that higher levels of physical activity are associated with better health-related quality of life [58,59,60,61]. Physically active adults are likely to have improved cardiovascular and metabolic health; better weight maintenance strategies; reduced risk of bone fracture and better bone mass and mineral density; increased muscular mass, strength, and power; and reduced risks of breast and colon cancer, diabetes, high blood pressure, coronary heart disease, stroke, hypertension, and depression [62,63,64,65,66,67,68,69].

Additionally, research on senescence has indicated that physical activity may play a vital protective role against the detrimental effects of age on psychological and physical health [70,71]. Higher physical activity in the elderly population has been linked to reduced prevalence of chronic conditions [72,73,74,75], decreased cognitive deterioration [76,77], improved physical health [74,78,79,80], better mental health [78,81,82], reduced mortality rates [74,75,83,84,85], and improved quality of life [78,81,82,86,87]. Furthermore, age-related cross-sectional studies have indicated that physically fit older participants (as opposed to their sedentary counterparts) had comparable results to younger participants on a range of cognitive tasks, abilities, and processes [88,89,90,91,92]. Similarly, longitudinal studies have indicated that involvement in moderate and vigorous physical activity may buffer against cognitive degeneration in older age [93]. Finally, intervention studies reveal that older adults who participated in physical activity programs showed improved cardiorespiratory fitness [94,95], increased heart rate variability and enhanced executive control [96], and improved cognitive performance [97]. 

## 3. Physical Activity and Gender 

It has been observed that there are systematic differences in the reasons males and females nominate for participating in physical activity [19,20,21]. Therefore, it is plausible to argue that gender differences exist in how individuals contrive and obtain benefits from engagement in specific physical activities, which may be functionally characterized by the benefits the activities seem to offer to those individuals. Despite this, recommended levels of physical activity around the world are more or less the same with minimal or no refinement related to gender [13]. 

Research has shown that males are more likely to be physically active across different life stages—through fetal and neonatal periods [98], infancy [99,100,101], childhood [102], adolescence [103], and adulthood [19,104]—as compared to their female counterparts. According to some researchers, this tilts the physical activity research and practice domains in favour of males, with health professionals often overlooking the requirements of females [105]. This is further exacerbated by the fact that females generally tend to engage less in physical activity [106], males take part in more specific sedentary behaviours [107], gender socialization and roles differ from birth onwards [108], and specific psychological, social, and environmental factors vary across lifetime [109,110], which play a significant role in mediating the relationship between gender and physical activity involvement. 

Although it is known that males and females tend to favour different kinds of physical activities, it is important to understand how this participation and consequent health outcomes are affected. Research indicates that level, intensity, type, and amount of physical activity involvement may have different beneficial effects for males and females. For instance, Asztalos and colleagues found that males participating in higher levels and vigorous intensity physical activity had lowered feelings of psychological distress, and females engaging in walking and moderate intensity physical activity had better emotional well-being and regulation [111]. This has also been confirmed in other studies that purport that males benefit from engaging in vigorous physical activities [112,113,114] whereas females benefit from low to moderate intensity physical activities [115]. 

Furthermore, studies involving differential intensity physical activity and health conditions show clear gender differences in terms of health outcomes. For instance, studies in cardiovascular disease [115,116,117,118] and diabetes [119] indicate that females are more likely than males to benefit from low to moderate intensity physical activity. In another study involving physical activity and cancer prevention, risk reduction in colon cancer in females was related to increased leisure time activity, whereas risk reduction in males was related to both leisure and occupational physical activity [120].

Gender differences in exercise habits also reveal that, with higher levels of exercise, the level of reported self-esteem increases in males and decreases in females [121]. In contrast, it has also been found that females who engage in low intensity physical activity report higher self-esteem and quality of life as compared to females who participate in high intensity physical activity [122]. This may be attributed to the different motives males and females have for engaging in physical activities. For instance, research suggests that males are more likely to participate in physical activity due to social and competitive reasons, whereas females are more likely to engage in physical activity for appearance motives [19,20,21]. 

## 4. Physical Activity across Atypical Population

While research on physical activity has established clear benefits for the healthy population [11,12,13], recent research has demonstrated that physical activity may also have positive implications for atypical conditions and population. For instance, it has been found that physical activity may aid in preventing obesity by helping individuals increase their total energy expenditure, decrease total body fat, and even build muscle mass by engaging in muscle-strengthening activities [123]. Furthermore, physical activity has been recommended as an alternative disease management strategy for individuals living with immunodeficiencies [124,125]. Additionally, research in the domain of cancer prevention suggests that physical activity may reduce the risk of a range of malignancies, including lung, colon, breast, prostate, and endometrial cancer [120,126,127,128,129,130,131,132]. Similarly, risk modeling studies indicate that physical activity may help reduce the risk of recurrent stroke [133]. Studies in diabetes have also indicated that physical activity, especially aerobic activity, is associated with lower cardiovascular and mortality risks in both type 1 and type 2 diabetes [134]. Research in different types of physical activity has also confirmed that it may play a vital role in improving overall physical health, including cardiorespiratory, musculoskeletal, and neuromotor fitness [135].

In recent years, the positive effects of physical activity on mental health have also come to the fore [136,137,138]. Physical activity has been found to be particularly effective in ameliorating the effects of stress, anxiety, and depression [139]. Asmundson and colleagues found that physical activity had positive therapeutic effects on stress and stress-related symptoms [137]. Similarly, systematic reviews of physical activity, especially aerobic and anaerobic exercise, suggest physical activity to be an efficacious transdiagnostic intervention for anxiety-related disorders [140]. Furthermore, randomized control trials have also indicated physical activity to be an effective tool in reducing symptoms of depression [141]. 

In addition to its psychophysiological benefits, physical activity has also been found to be positively associated with subjective health outcomes, including personal experiences of self-esteem, health behaviours, fitness, life situation, and ill-health [142,143]. A range of studies have reported that higher levels of physical activity were positively associated with higher self-esteem in children, adolescents, and young and middle-aged adults [144,145,146,147,148,149,150,151]. Finally, researchers have also found that physical activity not only fosters emotional, social, and motor skills, it also promotes personal well-being and strengthens relations between peers [152]. 

## 5. Physical Activity and Lifestyle

An individual’s capacity to undertake physical tasks of everyday living plays a vital role in their physical well-being and overall welfare. While higher physical functioning in individuals is associated with valued tasks and independent living, poor physical functioning has been found to be associated with poorer quality of life, inhibited social participation, and higher risk of death [153,154]. Other studies have also confirmed longitudinal and cross-sectional associations between physical functioning and physical activity [155]. 

Studies have indicated that physical functioning of individuals depends on a range of lifestyle and socio-economic factors and as such it is reasonable to believe that these factors may also play an important role in regulating involvement in physical activity. For instance, socio-economic status has been found to be one of the strongest predictors of physical functioning [156,157], with adults with lower socio-economic status found to be less likely to engage in healthy behaviours [158]. Furthermore, research indicates that higher levels of education, higher income, and living in affluent areas were positively associated with better physical functioning [159,160,161,162,163]. Additionally, a number of lifestyle-related behavioural risk factors, including smoking, excess alcohol consumption, and poor nutrition, have been found to negatively affect physical functioning [164,165,166,167,168,169,170]. 

## 6. Discussion

A review of the literature suggests that the benefits of engaging in regular physical activity has been consistently associated with a range of positive psychological and physical health outcomes for individuals across the life span. Research in the active living, sport, and exercise literature has reliably demonstrated that individuals may have different reasons for engaging in different forms of physical activities and as such may gain different benefits from engaging in those activities. For instance, it is vital for toddlers, pre-school, and school-aged children to engage in active play that fosters their movement, communication, confidence, social, and interaction skills. Therefore, in addition to the evident kinesiological benefits that they would obtain from engaging in physical activity, it would also be reasonable to state that while, on one hand, structured play can nurture their movement, intellectual, and problem-solving skills, unstructured play can also, on the other hand, cultivate their creative, imaginative, and social skills. Similarly, other studies have also indicated that older adults tend to engage in leisure time physical activities in order to relax, maintain independence, flexibility, and mobility, and reduce muscle atrophy, amongst other reasons. Despite this, majority of research in the sport and exercise domain has focused exclusively on the type, intensity, frequency, and/or amount of physical activity with no or very little regard given to the saliency of other influences which may play an equivale role in underscoring individuals’ primary participation in those activities. Health professionals and policy makers should, therefore, focus on developing targeted interventions that not only accentuate physiological benefits in physical activity participation, but also emphasize on the motivational and enjoyable aspects of physical activity involvement with especial attention given to individual-activity fit. Designing novel and tailored evidence-based interventions specific to participants would ensure that those individuals engage in appropriate forms of physical activities, which would maximize satisfaction and reduce drop-out rates. Furthermore, creating smart and innovative campaigns will not only promote the uptake of physical activity, but will also aid professionals in understanding and addressing barriers in an effective and efficient manner.

Physical activity is a complex behaviour which often has personal, social, and public antecedents and consequences. Individuals may engage in physical activity in many ways, with multiple combinations of dose-responses, motives, contexts, and outcomes. It is, therefore, imperative to understand what constitutes physical activity engagement, how it is perceived by individuals, and how it gets manifested in everyday life. Future research in this domain must focus on ethnographic, narrative, intervention, and longitudinal studies to complement quantitative experiments in order to arrive at a comprehensive conceptualization of physical activity involvement along with its practical implications. This must also include how much physical activity is adequate, sufficient, or recommended for individuals with specific needs or goals. 

Physical activity may also have varied meanings for different individuals depending on the context, lifestyle choices, religious and spiritual practices, and local customs and traditions, in which the activities are being carried out. Given the multivariate nature of physical activity, exploratory and cross-sectional studies ought to focus on social, linguistically-diverse, and communal components of physical activity involvement. This may, for instance, include school-based physical education programs, or slow body-movement intervention programs in aged care setting, or appropriate diet-exercise awareness campaigns to enable better decision-making in clients. Similarly, public health announcements, messages, and campaigns should be clear, updated regularly, and modernized. 

Given the efficaciousness of physical activity across the life span, another area that would particularly benefit is the health care sector. There is an insistent need for researchers, practitioners, consultants, and policy makers to formulate a unified diagnostic and prescriptive code by incorporating physical activity across a variety of settings, whereby physicians could potentially counsel and recommend clients appropriate physical activity as part of their treatment plans to further therapeutic goals. This would not only help clients gain valuable kinesiological benefits but also address excessive dependence on medications that is prevalent across numerous health care settings and conditions. This also means that appropriate training and study modules must be incorporated in educational and professional settings to equip future practitioners with relevant and necessary skills and knowledge. 

With the advent and excessive use of technology in everyday life, future research could also focus on utilizing mobile applications, web-based platforms, and virtual or augmented reality wearables to help individuals keep track of their physical activity goals, maintain adherence, and gain desired health outcomes. This could also be used to understand pertinent shifts in generational differences, social and community trends, and personal preferences. Finally, utilizing both traditional and novel methodologies may ultimately assist us in understanding why individuals engage in different forms of physical activity and the myriad benefits they could achieve from such engagement. This will undoubtedly have huge implications for the psychological and kinesiological health of individuals. 

## 7. Conclusions

In summary, it is now widely recognized that engagement in appropriate forms of physical activity may have numerous psychological, kinesiological, and social benefits. It has been noted in the literature that people tend to engage in diverse forms of physical activity for primary participation reasons by virtue of their age, gender, condition, and context. Understanding the influences of these variables and tailoring appropriate programs and interventions will greatly assist individuals maximize their physical activity needs and goals. 
